# Identification of tubulointerstitial genes and ceRNA networks involved in diabetic nephropathy via integrated bioinformatics approaches

**DOI:** 10.1186/s41065-022-00249-6

**Published:** 2022-09-26

**Authors:** Haiyan Cao, Xiaosheng Rao, Junya Jia, Tiekun Yan, Dong Li

**Affiliations:** 1 Department of Nephrology, Tianjin Medical University General Hospital, Tianjin, 300052 China; 2 Department of Urology, The First Affiliated Hospital of Guangzhou Medical University, Guangzhou, 510120 China

**Keywords:** Diabetic nephropathy, Integrated bioinformatics approaches, Tubulointerstitial, Genes, ceRNA networks

## Abstract

**Background:**

Diabetic nephropathy (DN) is the major cause of end-stage renal disease worldwide. The mechanism of tubulointerstitial lesions in DN is not fully elucidated. This article aims to identify novel genes and clarify the molecular mechanisms for the progression of DN through integrated bioinformatics approaches.

**Method:**

We downloaded microarray datasets from Gene Expression Omnibus (GEO) database and identified the differentially expressed genes (DEGs). Enrichment analyses, construction of Protein–protein interaction (PPI) network, and visualization of the co-expressed network between mRNAs and microRNAs (miRNAs) were performed. Additionally, we validated the expression of hub genes and analyzed the Receiver Operating Characteristic (ROC) curve in another GEO dataset. Clinical analysis and ceRNA networks were further analyzed.

**Results:**

Totally 463 DEGs were identified, and enrichment analyses demonstrated that extracellular matrix structural constituents, regulation of immune effector process, positive regulation of cytokine production, phagosome, and complement and coagulation cascades were the major enriched pathways in DN. Three hub genes (CD53, CSF2RB, and LAPTM5) were obtained, and their expression levels were validated by GEO datasets. Pearson analysis showed that these genes were negatively correlated with the glomerular filtration rate (GFR). After literature searching, the ceRNA networks among circRNAs/IncRNAs, miRNAs, and mRNAs were constructed. The predicted RNA pathway of NEAT1/XIST-hsa-miR-155-5p/hsa-miR-486-5p-CSF2RB provides an important perspective and insights into the molecular mechanism of DN.

**Conclusion:**

In conclusion, we identified three genes, namely CD53, CSF2RB, and LAPTM5, as hub genes of tubulointerstitial lesions in DN. They may be closely related to the pathogenesis of DN and the predicted RNA regulatory pathway of NEAT1/XIST-hsa-miR-155-5p/hsa-miR-486-5p-CSF2RB presents a biomarker axis to the occurrence and development of DN.

## Introduction

Diabetic nephropathy (DN) is a destructive complication of diabetes mellitus and a major cause of end-stage renal disease [1]. As an overwhelming majority of the renal parenchyma, the tubulointerstitial compartment plays an irreplaceable role in the pathogenesis of DN. Representative tubulointerstitial changes of DN include the hypertrophy of the renal tubular, thickening of the tubular basement membrane, and the fibrosis of the renal interstitial, which promote the development of DN [2, 3]. Some emerging biological pathways, such as oxidative stress, transforming growth factor-β activation, proinflammatory cytokines, and chemokines, are also involved in tubulointerstitial lesions of DN [4]. Since its complex pathogenesis, exploring potential genes and mechanisms could provide novel insight into the understanding of DN.

In the last few years, much attention has been paid to the integrated analysis of transcriptomic and microarray due to its widespread use in multiple diseases, including cancers and non-cancers. The previous explorations combined with bioinformatics have revealed several biomarkers in DN, such as Protein S and COL4A3 [5, 6]. The presence of microalbuminuria has been considered a well-known biomarker to indicate the occurrence of the advanced DN [7]. Whereas microalbuminuria cannot sufficiently predict the DN patients with non-proteinuria or with slight pathophysiological changes, exploring extra biomarkers of tubulointerstitial lesions to indicate the early stage of DN seems indispensable. Furthermore, competitive endogenous RNA (ceRNA) networks that based on the interactions among the main non-coding RNAs (ncRNAs), including microRNAs (miRNAs), long non-coding RNAs (IncRNAs), and circular RNAs (circRNAs), are capable of providing a significant opportunity to advance the understanding of biomarkers and mechanisms that closely related to DN.

Our present work identified the difference in the mRNA expression profiles between DN samples and controls from human tubulointerstitial tissues. The limma package was used to recognize differentially expressed genes (DEGs). Gene set enrichment analysis (GSEA), Gene Ontology (GO), and Kyoto encyclopedia of genes and genomes (KEGG) Pathways were performed to explore the mechanisms that impact the pathogenesis of DN. The Protein–protein Interaction (PPI) network was constructed to obtain hub genes. Validation datasets were further used to validate the expression and plot the ROC curve of hub genes. The clinical features of the hub genes were explored. Based on the interaction relationship between the predicted miRNAs, IncRNAs, and circRNAs, we constructed ceRNA networks to elucidate a novel mechanism for accelerating DN progression in transcriptional networks. The identified hub genes and potential ceRNA networks are expected to provide insights into the molecular mechanism of tubulointerstitial lesions in DN.

## Materials and methods

### Data sources

We screened relevant gene expression datasets from the Gene Expression Omnibus (GEO) genomics data repository (http://www.ncbi.nlm.nih.gov/geo) using diabetic nephropathy and tubulointerstitial as the keywords [8]. The screening criteria are set as follows: the microarray expression profile should include human tubulointerstitial samples and provide complete raw data. Eventually, we selected the microarray dataset (GSE30529) that was downloaded from the Affymetrix GPL571 platform (Affymetrix Human Genome U133A 2.0 Array) as the test dataset. GSE30529 was the subseries of GSE30122, from which we collected 10 DN samples (GSM757014-GSM757023) and 12 controls (GSM757024-GSM757035) of human tubulointerstitial tissues.

### Quality control and identification of DEGs

We used GEOquery (version 2.54.1) and limma package (version 3.42.2) in R software (version 3.6.3) to read data and complete the analysis of DEGs, respectively. Data normalization and log2 conversion were included in the pre-processed analysis. Probe sets with no relevant gene symbols or gene symbols with an excess of one probe set were removed or averaged. To evaluate the quality of the selected dataset, we analyzed the processed data boxplot, principal component analysis (PCA), and uniform manifold approximation and projection (UMAP). We considered these genes with adj. *P*-value < 0.05 and |logFC (fold change)|> 1 as DEGs. We further used the ggplot2 (version 3.3.3) package and ComplexHeatmap (version 2.2.0) package of R software to draw the volcano and heatmaps of the acquired DEGs [9].

### Enrichment analyses

To evaluate the association between gene sets and biological signals, we performed the GSEA analysis [10]. After downloading GSEA_4.1.0 and c5: GO gene sets (c5.all.v7.1.symbols. gmt), we analyzed it via the clusterProfiler package (version 3.14.3) in R software [11]. The threshold value of |Normalized Enrichment Score (NES)|> 1, False Discovery Rates (FDR) < 0.05, and *p*-value < 0.05 were considered statistically significant in GSEA. GO and KEGG enrichment analyses of identified DEGs were annotated with clusterProfiler package (version 3.14.3) and visualized by ggplot2 (version 3.3.3). GO analysis can provide scientists with the better understanding of the functional annotations of the gene products [12]. KEGG Pathway is a tool to help biologists understand the systemic functions of the cell and even the organism [13]. Q value < 0.05 and gene count ≥ 2 were considered to indicate statistically significant results.

### PPI network construction and significant module analysis

STRING (version 11.0), an online database that can explore the underlying relationship among proteins, was used to construct the complex PPI network with a combined score > 0.7 [14]. Cytoscape (version 3.8.2) was used to visualize the PPI network [15]. Minimal Common Oncology Data Elements (MCODE) (version 2.0.0), a plugin of Cytoscape, was used to construct significant modules with the filter criteria: degree cutoff = 2, max depth = 100, K-core = 2, and node score cutoff = 0.2.

### Identification of hub genes and prediction of target miRNAs

We used cytoHubba (version 0.1), a plugin of Cytoscape software, to obtain hub genes by acquiring the interacted genes of the top 10 genes identified by the following algorithm modules, namely Maximal Clique Centrality (MCC), Density of Maximum Neighborhood Component (DMNC), Maximum Neighborhood Component (MNC), and Degree [16]. Three online tools (miRmap, microT, miRanda) were applied to predict target miRNAs of hub genes, and we chose the miRNAs that appeared more than once in searching tools as target miRNAs. Given the regulatory relationship between the hub genes (mRNA) and their target miRNAs, we used Cytoscape software to perform the co-expressed network of mRNAs-miRNAs.

### Validation and ROC curve analysis of hub genes by another GEO dataset

The GEO dataset (GSE104954) was recognized as the validation dataset with selected 7 DN samples (GSM2811029-GSM2811035) and 18 controls (GSM2811043-GSM2811060) from human tubulointerstitial tissues. We used the validation dataset to verify the mRNA expression and plot the ROC curve of the hub genes by the ggplot2 package (version 3.3.3). The statistical method of t-test was used to compare the differences between the DN samples and controls, and we considered *P*-value < 0.05 as significant.

### Construction of ceRNA networks

StarBase (http://starbase.sysu.edu.cn/index.php) is a novel database developed to accelerate the comprehensive understanding of miRNA-target intersection maps based on the CLIP-Seq and Degradome-Seq data [17]. We used StarBase to explore the interactive relationships between the IncRNAs, circRNAs, and specific miRNAs with the screening criteria of mammalian, human, h19 genome, strict stringency (> = 5) of CLIP-Data, with or without degradome data, and with or without data of pan-cancer. Finally, the intersected IncRNAs and circRNAs were recognized as the final target results of certain miRNAs. The ceRNA networks among mRNAs, miRNAs, IncRNAs, and circRNAs were constructed and visualized by the Cytoscape software.

### Clinical and statistical analysis

The data from Nephroseq v5 online platform (http://v5.nephroseq.org) was extracted to explore clinical characteristics of gene expression in human tubulointerstitial tissues [18]. We performed a correlation analysis of glomerular filtration rate (GFR) [19], proteinuria [20], and age in tubulointerstitial samples of DN patients and compared gene expression between the subnephrotic proteinuria group and nephrotic proteinuria group [20]. A statistical method of Student’s t-test was used to analyze the data between the two groups. The cutoff criterion was set as *p* < 0.05. Insignificant results are not displayed.

## Results

### Quality control and identification of DEGs

**Fig. 1:**
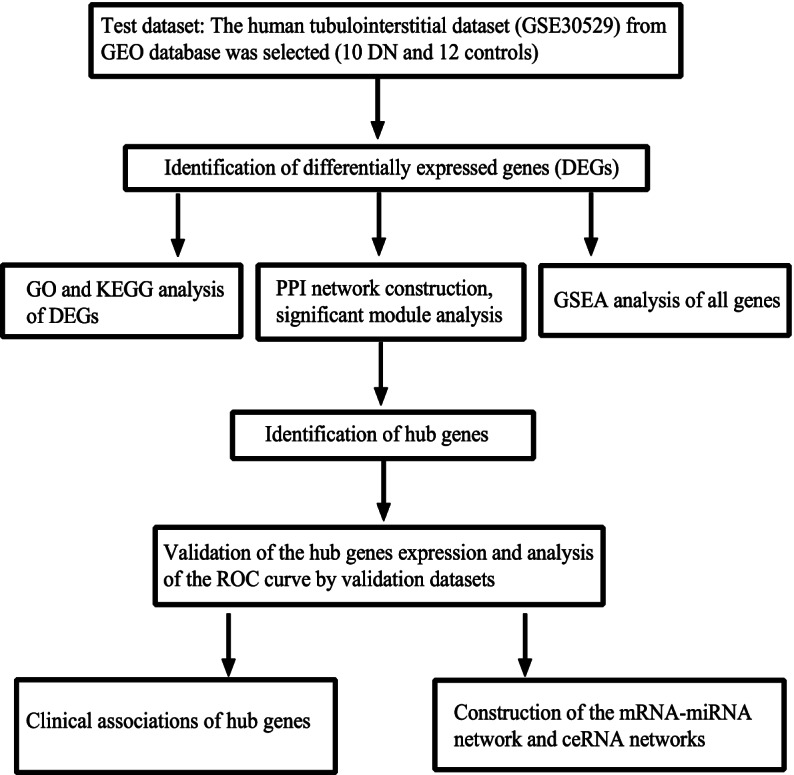
The flowchart of this study

**Fig. 2:**
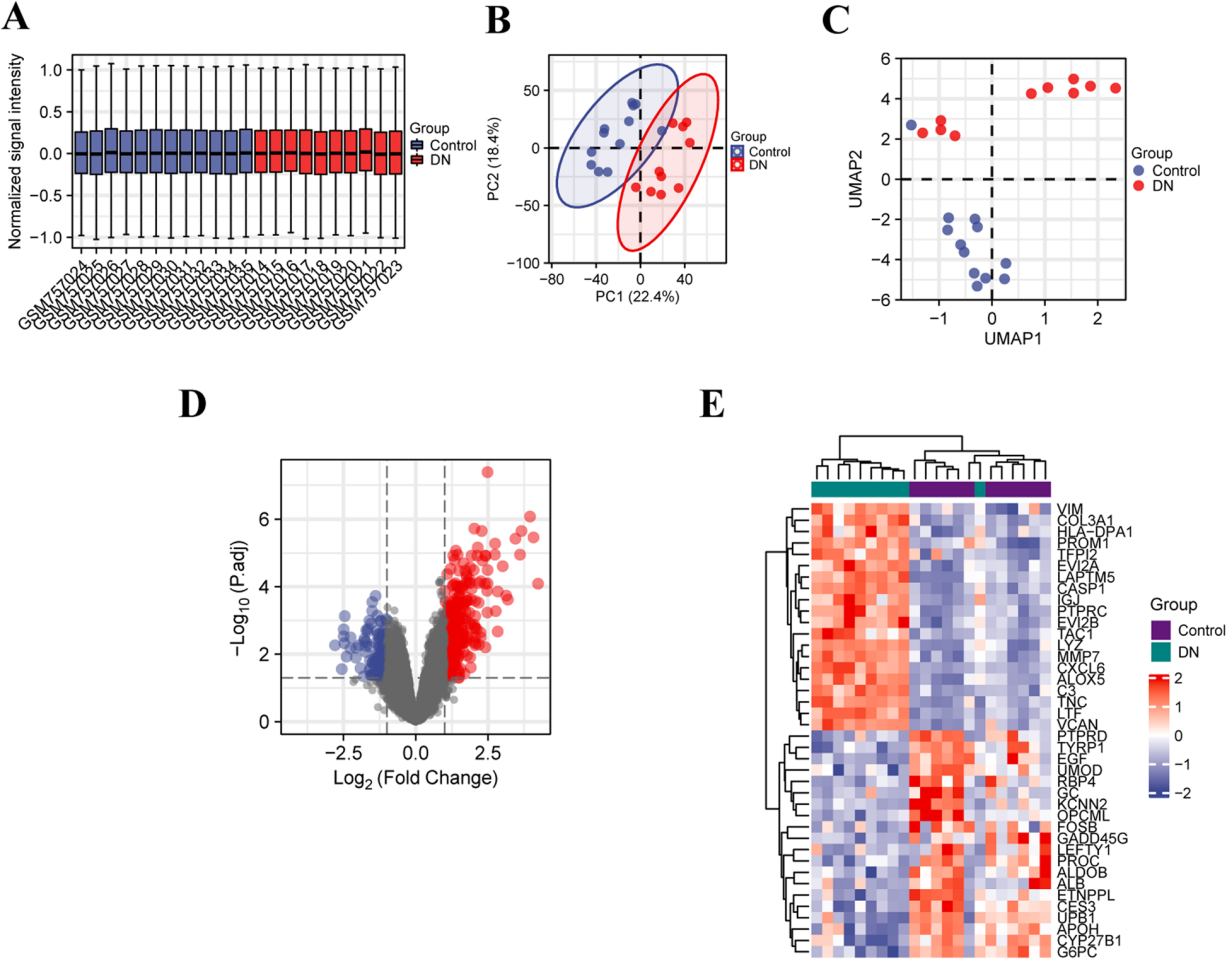
Quality control and Identification of DEGs. **A** The processed data boxplot. **B** The PCA plot. **C** The UMAP plot. **D** The valcano plot of DEGs. **E** The heatmap of the top 20 significant up-regulated and down-regulated DEGs. DN: diabetic nephropathy; DEGs: differentially expressed genes; PCA: principal component analysis; UMAP: uniform manifold approximation and projection

Figure 1 shows the overall flowchart of this present research. The processed data boxplot displayed that the medians of each sample were almost on the same line (Fig. 2A). The PCA and UMAP plots suggested a good distinction between DN and control samples (Fig. 2B, C). After downloading the tubulointerstitial data from GSE30529, we recognized 463 DEGs that consist of 340 up-regulated DEGs and 123 down-regulated DEGs. Figure 2D and E showed the volcano map and heatmap of the DEGs.

### Analysis of the functional characteristics

**Fig. 3:**
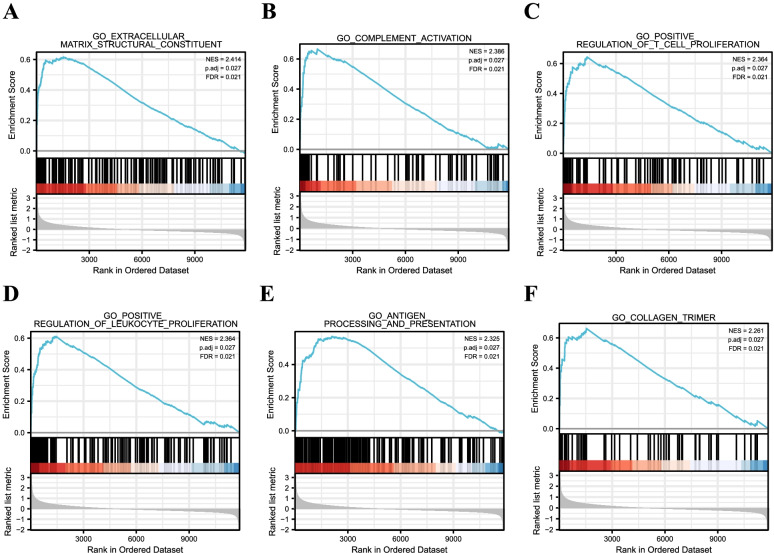
GSEA plot showed the most enriched gene sets in DN. **A** The most signifcant enriched gene set was extracellar matrix structural constituent (ES = 0.617, NES = 2.414, *p* < 0.05). **B** The second signifcant gene set was complement activation (ES = 0.666, NES = 2.386, *p* < 0.05). **C** The third signifcant enriched gene set was positive regulation of T cell production (ES = 0.644, NES = 2.364, *p* < 0.05). **D** The fourth signifcant enriched gene set was positive rgulation of leukocyte proliferation (ES = 0.612, NES = 2.364, *p* < 0.05). **E** The fifth signifcant enriched gene set was antigen processing and presentation (ES = 0.570, NES = 2.325, *p* < 0.05). **F** The sixth signifcant enriched gene set was collagen trimer (ES = 0.662, NES = 2.261, *p* < 0.05). The defined criteria for significant gene sets were |Normalized Enrichment Score (NES)|> 1, False Discovery Rates (FDR) < 0.05 and *p*-value < 0.05. DN: diabetic nephropathy; ES: enrichment score; NES: normalized enrichment score

**Fig. 4:**
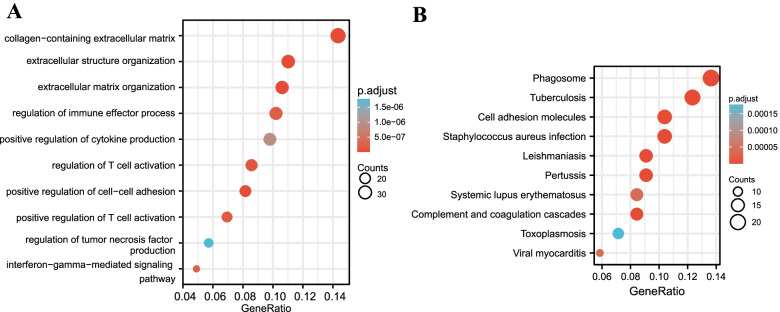
Enrichment analysis of DEGs. **A** The bubble plot showing the top 10 enriched biological processes of DEGs. **B** The bubble plot showing the top 10 enriched pathways of KEGG in DN samples. DN: diabetic nephropathy

To evaluate GO analysis of all genes, we performed GSEA analysis. We found that the most enriched gene sets were associated with extracellular matrix structural constituent, complement activation, and positive regulation of T cell proliferation in DN samples (Fig. 3). We subsequently performed the GO and KEGG pathway analyses of DEGs. Compared to the controls, DN patients are significantly enriched in the extracellular matrix and inflammatory response, including collagen-containing extracellular matrix, extracellular structure organization, and regulation of immune effector process (Fig. 4A). Based on the KEGG pathways, the top 10 enriched pathways involved Phagosome, Staphylococcus aureus infection, Pertussis, Leishmaniasis, Tuberculosis, Complement and coagulation cascades, Cell adhesion molecules, Viral myocarditis, Systemic lupus erythematosus, and Toxoplasmosis (Fig. 4B).

### PPI network construction and module analysis

**Fig. 5:**
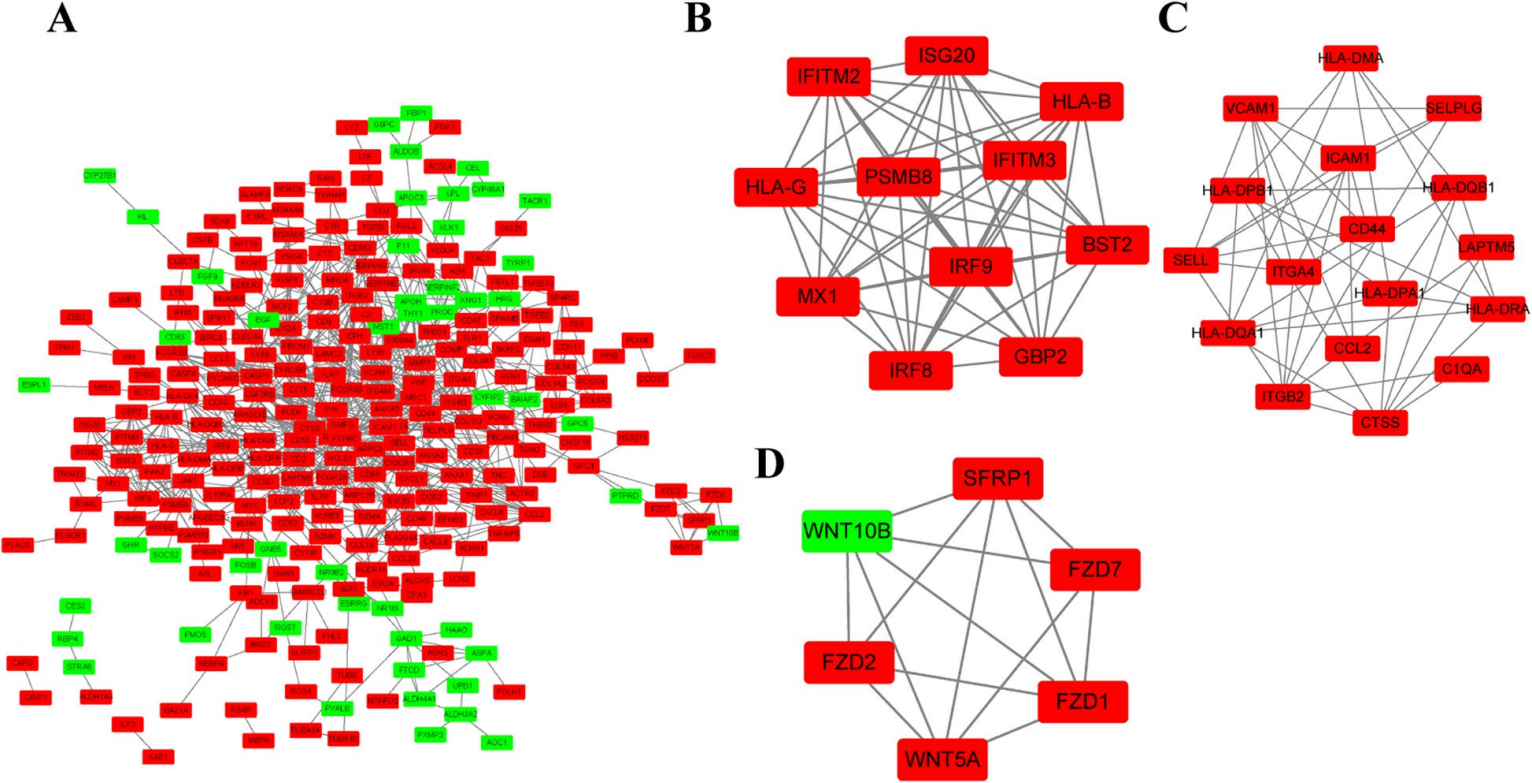
PPI network and three significant modules. **A** The PPI network includes 277 nodes and 803 edges. **B-D** three modules are extracted with MCODE score > 5. Red nodes indicate up-regulated DEGs and green nodes indicate down-regulated DEGs. PPI: protein–protein interaction

The complex PPI network was constructed with 277 nodes and 803 edges in Fig. 5A. Through plugin MCODE of Cytoscape, we recognized 3 significant modules with an MCODE score > 5. Module one had the highest score with 11 (11 nodes and 55 edges), module two had the second-highest score with 5.625 (17 nodes and 45 edges), followed by module three had the third-highest score with 5.60 (6 nodes and 14 edges) (Fig. 5B-E).

### Identification of hub genes and prediction of target miRNAs

**Fig. 6:**
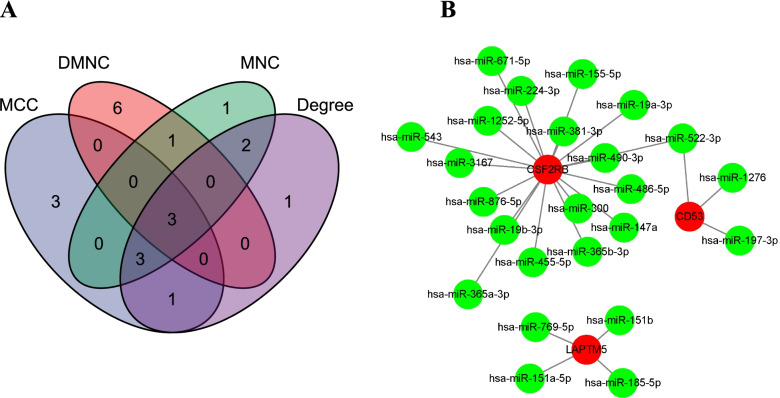
Identification of hub genes and the co-expressed network of mRNAs-miRNAs. **A** The Venn diagram of three hub genes screened by four algorithms. **B** The mRNA-miRNA network was visualized by Cytoscape. CSF2RB had 18 target miRNAs, CD53 had 3 target miRNAs, and LAPTM5 had 4 target miRNAs. Red circles represented mRNAs and green circles represented miRNAs

Four algorithms were used to identify the hub genes via the Venn diagram. As a result, 3 intersected genes were obtained (Fig. 6A). Table 1 lists the hub genes together with their full name and descriptions. We then explored 24 target miRNAs of 3 hub genes based on the regulatory relationship between mRNAs and miRNAs. There are 25 mRNA-miRNA pairs in the co-expressed mRNA-miRNA network (Fig. 6B).

**Table 1:** The details of the intersected hub genes

Gene symbol	Full name	Description
CD53	CD53 Molecule	CD53 is a member of the transmembrane 4 superfamilies. It contributes to the transduction of CD2-generated signals in T cells and natural killer cells and has been suggested to play a role in growth regulation
LAPTM5	Lysosomal Protein Transmembrane 5	LAPTM5 is a transmembrane receptor that is associated with lysosomes
CSF2RB	Colony Stimulating Factor 2 Receptor Subunit Beta	CSF2RB is a high-affinity receptor for interleukin-3, interleukin-5, and granulocyte–macrophage colony-stimulating factor

### Validation and ROC curve of hub genes

**Fig. 7:**
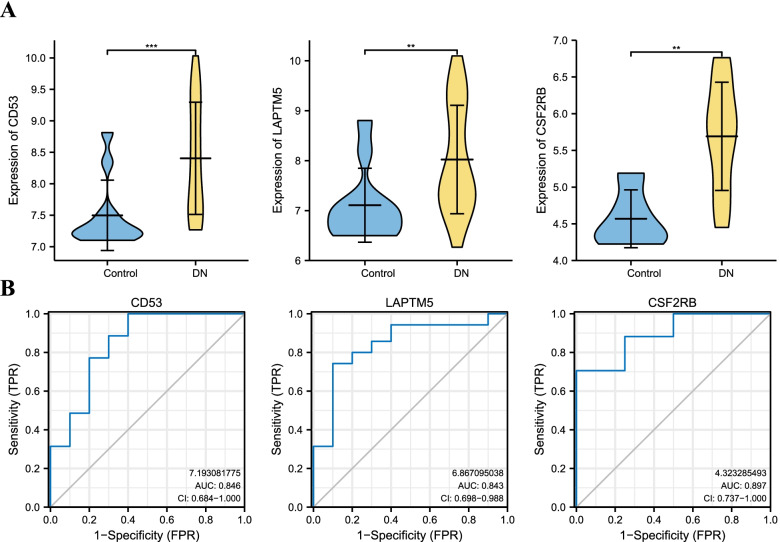
Verification and ROC curve analysis of the hub genes by another human tubulointerstitial dataset. **A** CD53, LAPTM5, and CSF2RB are up-regulated in DN samples compared with controls. ***: *p* < 0.001, **: *p* < 0.01, *: *p* < 0.05. **B** CSF2RB has the highest score (AUC value: 0.897), CD53 has the second highest score (AUC value: 0.846) and LAPTM5 has the third highest score (AUC value: 0.843). ROC: Receiver Operating Characteristic; AUC: Area under the curve; DN: diabetic nephropathy

To assess the reliability of these hub gene expressions, we used the validation dataset to explore the mRNA levels of the obtained hub genes. The results displayed that 3 hub genes showed up-regulated mRNA levels in DN (Fig. 7A). To further evaluate the sensitivity and specificity of the hub genes, we analyzed the ROC curve with the validation dataset. Among the results, CSF2RB had the highest areas under the curve (AUC) score ( AUC value: 0.897), CD53 had the second-highest AUC score ( AUC value: 0.846), followed by LAPTM5 had the third-highest AUC score ( AUC value: 0.843) (Fig. 7B).

### Clinical analysis

**Fig. 8:**
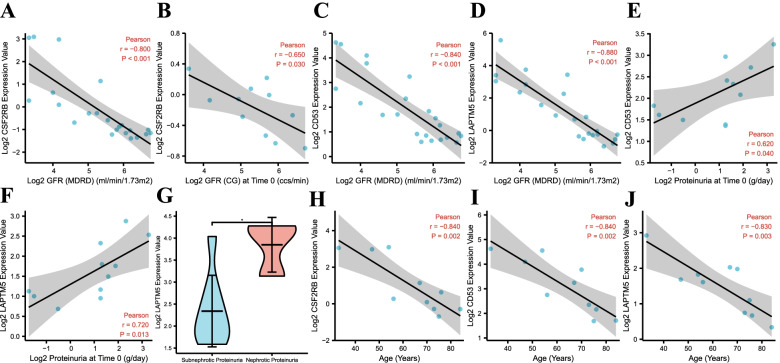
Explorations between clinical features and hub genes (CSF2RB, CD53 and LAPTM5) in human tubulointerstitial samples. **A** The mRNA expression of CSF2RB was negatively correlated with GFR (MDRD). **B** The mRNA expression of CSF2RB was negatively correlated with GFR (CG). **C** The mRNA expression of CD53 was negatively correlated with GFR (MDRD). **D** The mRNA expression of LAPTM5 was negatively correlated with GFR (MDRD). **E** The mRNA expression of CD53 was positively correlated with proteinuria. **F** The mRNA expression of LAPTM5 was positively correlated with proteinuria. **G** The mRNA expression of LAPTM5 in nephrotic proteinuria group was higher than that of subnephrotic proteinuria group. **H** The mRNA expression of CSR2RB was negatively correlated with age. **I** The mRNA expression of CD53 was negatively correlated with age. **J** The mRNA expression of LAPTM5 was negatively correlated with age. GFR: glomerular filtration rate; MDRD: modification of diet in renal disease; CG: Cockcroft Gault; *: *p* < 0.05; *P* < 0.05 was considered significant

Based on the Nephroseq v5 platform, we investigated the possible impact of the hub genes in tubulointerstitial samples in DN. Correlation analyses showed that the mRNA levels of CSF2RB, CD53, and LAPTM5 in human tubulointerstitial samples were negatively correlated with GFR **(**Fig. 8A-D**)**, indicating that these hub genes may accelerate the tubulointerstitial lesions of DN. Then, the mRNA levels of CD53 and LAPTM5 in human tubulointerstitial samples had positive correlations with proteinuria (Fig. 8E, F), further suggesting that CD53 and LAPTM5 may be involved in the progression of tubulointerstitial injuries of DN. Additionally, we found that, in contrast with the subnephrotic proteinuria group, the mRNA expression level of LAPTM5 in renal tubulointerstitial samples was higher in the nephrotic proteinuria group (Fig. 8G). Furthermore, the mRNA levels of CSF2RB, CD53, and LAPTM5 were conversely related to their age (Fig. 8H, J).

### Construction of ceRNA networks

It is well known that miRNAs can inhibit mRNA translation or lead to mRNA degradation by binding to target mRNAs, thus achieving the regulation of gene expression. However, the ceRNA hypothesis interpreted that IncRNAs, circRNAs, and pseudogenes that act as competing endogenous RNAs or natural microRNA sponges can increase gene expression by binding microRNA response elements (MREs) [21]. Based on the Starbase platform, we predicted corresponding IncRNAs and circRNAs of target miRNAs. Finally, we selected the IncRNAs, circRNAs that appeared in most of the forecasted results of target miRNAs to be our final IncRNAs, circRNAs. Of the predicted results in the Starbase tool, we regarded the circRNAs that qualified with the highest score or /and the most samples in the circBase database to be the eventual target circRNAs since one transcript corresponds to multiple shear sites of circRNA.

**Fig. 9:**
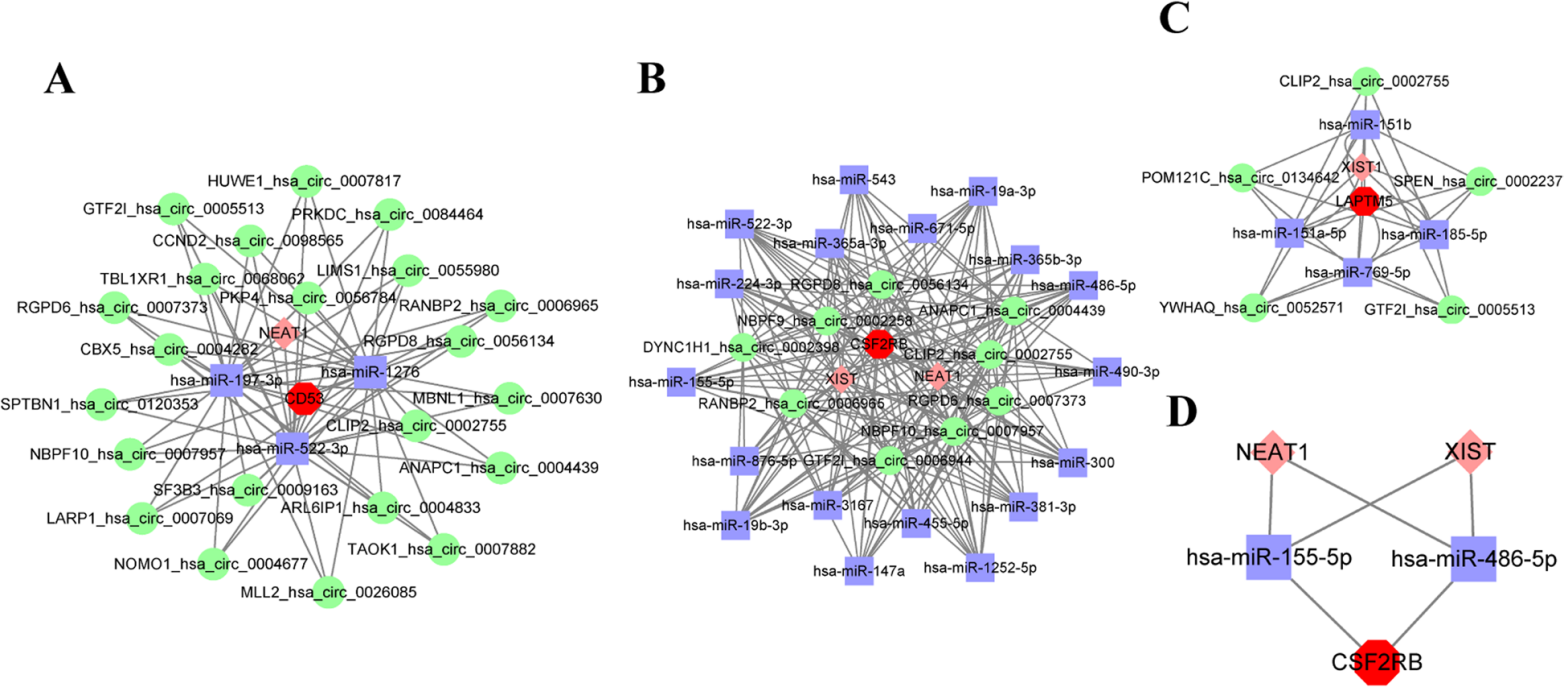
Three ceRNA networks of CD53, CSF2RB and LAPTM5 and the potential RNA regulatory pathways. **A** The ceRNA network of CD53. **B** The ceRNA network of CSF2RB. **C** The ceRNA network of LAPTM5. **D** NEAT1/XIST-miR-155-5p/miR-486-5p-CSR2RB. Red octagons note the hub genes, purple rectangles note miRNAs, pink diamonds note IncRNAs, and syan circles note circRNAs

Just as the forecasted results of circRNAs and IncRNAs (Fig. 9A, B, C), we acquired 22 circRNAs and 1 IncRNA of 3 miRNAs of CD53, which were paired into 66 circRNA-miRNA interactions, 3 IncRNA-miRNA interactions, and 3 miRNA-mRNA interactions; 9 circRNAs and 2 IncRNAs of 18 miRNAs of CSF2RB, which were paired into 135 circRNA-miRNA interactions, 18 IncRNA-miRNA interactions, and 29 miRNA-mRNA interactions; and 5 circRNAs and 1 IncRNA of 4 miRNAs of LAPTM5, which were paired into 20 circRNA-miRNA interactions, 4 IncRNA-miRNA interactions, and 4 miRNA-mRNA interactions. After an extensive literature search, we chose two reported down-regulated miRNAs and two reported up-regulated IncRNAs in DN, for further analyses. We speculated that the RNA regulatory pathway of NEAT1/XIST-hsa-miR-155-5p/hsa-miR-486-5p-CSF2RB might play an essential role in DN (Fig. 9D).

## Discussion

DN is the major cause of renal failure across the world. The pathogenetic mechanisms of the tubulointerstitium, including the activation of intracellular signals [1], adverse changes secondary to the glomerulus, and hypoxia-induced lesions [22, 23], have emerged as a critical part of the occurrence and progression of DN. Although a great deal of effort has been undertaken so far, we still lack a thorough understanding of the pathogenesis of DN. Fortunately, this emerging combination of high-throughput microarray technology and bioinformatics methods allows us to identify important genes and biological pathways associated with tubulointerstitial lesions in DN and gain insights into the pathogenesis of DN.

Totally 12,548 genes are included in the GSEA enrichment analysis, and 463 DEGs are included for GO and KEGG analyses. GSEA and GO enrichment analyses both suggested that extracellular matrix (ECM) structural constituent, ECM organization, collagen-containing ECM, and extracellular structure organization were highly enriched in DN groups. The hallmark of the pathogenesis of DN is an increased ECM accumulation, and the ECM levels are usually regulated by a balance between the deposition and degradation of the ECM components [24]. The deposition of ECM proteins, including collagen I, decorin, and biglycan, are all involved in tubulointerstitial fibrosis of DN [25, 26]. As significant endopeptidases for degrading ECM protein components, matrix metalloproteinases (MMPs) can accelerate diverse physiologic and pathological damages in DN [27]. In human DN, MMPs dysregulation interferes with ECM degradation and hydrolysis and is involved in an established renal injury [28].

Additionally, GO and KEGG pathway analyses were also enriched in regulation of immune effector process, positive regulation of cytokine production, phagosome, complement and coagulation cascades, and cell adhesion molecules. The inflammatory response is a mechanism induced by activated stimuli to provide protection, while non-resolving inflammation triggers the collateral damage of tissues and organisms [29]. Under high glucose conditions, increasing evidence points out the critical role of inflammation response in the development and progression of DN [30]. The activation of the complement system, deposition of immunoglobulin or immunoglobulin complexes, and activation of macrophages are all involved in the pathogenesis of DN [31–33].

After constructing the PPI network, we acquired three hub genes ( CSF2RB, CD53, and LAPTM5). Further validation suggested that they showed up-regulated expressions and had high AUC values. Combined with their negative correlations with GFR, we speculated that CSF2RB, CD53, and LAPTM5 might be the key genes in human tubulointerstitial lesions of DN. The subsequent mRNA-miRNA network and ceRNA networks were accomplished to offer the opportunity to understand the mechanisms of DN from the transcriptomic level.

CSF2RB(colony-stimulating factor 2 receptor β subunit) is the common subunit of receptors for interleukin 3 (IL3), GM-CSF, and IL5 and is responsible for the initiation of signal transduction triggered by ligand binding [34]. Considerable evidence has revealed that IL3, GM-CSF, and IL5 regulate multiple inflammatory responses that contribute to the pathology in chronic inflammation [35, 36]. Since inflammatory responses are the key contributors to the development of DN [37], we speculated that CSR2RB has a critical role in the molecular mechanisms of DN. Our work found that CSF2RB expressed an increased mRNA level in human tubulointerstitial samples of DN and had a high diagnostic value (AUC = 0.897). Furthermore, the level of CSF2RB in human renal tubulointerstitial samples was conversely correlated with GFR, and we thus regarded CSR2RB as a key gene in the progression of tubulointerstitial lesions in DN.

CD53, a component of the tetraspanin family member expressed in the immune compartment, has been reported to regulate integrin-related function by adjusting integrin signaling pathways and changing the general distribution of integrins located on the immune cell surface [38, 39]. As known that relevant biological pathways about integrins have been recognized as an essential factor in the pathogenesis of DN [19], we then supposed that CD53 might play potential roles in DN. In our study, we investigated that CD53 showed an up-regulated level in DN samples. This level had a negative correlation with GFR and a positive correlation with proteinuria in human tubulointerstitial samples of DN, further illustrating the impact of CD53 involved in tubulointerstitial lesions of DN.

LAPTM5 (lysosomal-associated protein transmembrane 5) can exert positive proinflammatory effects in macrophages through activating NF-κB and MAPK signaling pathways, promoting the production of proinflammatory cytokines [40]. The accumulation of macrophages in diabetic kidneys has been proven to associate with DN progression as this accumulation is closely related to interstitial myofibroblast accumulation and interstitial fibrosis scores [41–44]. In our study, we found that LAPTM5 was augmented in DN, and this level was negatively correlated with GFR and actively correlated with proteinuria in human tubulointerstitial samples of DN. Combined with the results mentioned earlier and its diagnostic value (AUC value:0.843), we regarded LAPTM5 as a hub gene in DN.

Based on the predicted target miRNAs, IncRNAs, and circRNAs, we subsequently visualized the ceRNA networks by Cytoscape software. After a literature search for target miRNAs and IncRNAs, the down-regulated miRNAs and up-regulated IncRNAs were chosen for further analysis. Among them, hsa-miR-155-5p and hsa-miR-486-5p were down-regulated in DN samples [45, 46]. Additionally, IncRNAs of Nuclear Enriched Abundant Transcript-1 (NEAT1) and X-inactive specific transcript (XIST) have emphasized their high expression level in DN. Under hyperglycemia, the expression of NEAT1 was up-regulated in DN and led to renal tubular epithelial-mesenchymal transition (EMT) and renal fibrosis [47]. Another IncRNA of XIST also showed an up-regulated level in human DN, and this increased expression of XIST is closely related to renal fibrosis [48]. Based on the proposed ceRNA hypothesis, these observations may support the possibility that the regulatory pathway of NEAT1/XIST-hsa-miR-155-5p/hsa-miR-486-5p-CSF2RB might be involved in the molecular mechanisms of DN complicated with tubulointerstitial lesions.

Nevertheless, our article still has some limitations. First, this is a retrospective study that may command Vivo experiments with a larger sample size to prove our observations; Second, the definite function of the hub genes and the molecular mechanisms under DN needs to be further verified in future investigations before being applied in clinical practice, which will be the main direction of our future work.

## Conclusion

Our research identified three genes, namely CSF2RB, CD53, and LAPTM5, as the tubulointerstitial genes of DN and helped us further elucidate the molecular mechanisms of DN at the transcriptome level. The hypothetical pathway of NEAT1/XIST-hsa-miR-155-5p/hsa-miR-486-5p-CSF2RB may play a vital role in the progression of tubulointerstitial lesions implicated in early DN.

## Data Availability

The datasets presented in this study can be found in Gene Expression Omnibus (https://www.ncbi.nlm.nih.gov/geo/). The names of the repository/repositories and accession number(s) can be found in the article.

## Abbreviations

ceRNA: Competitive endogenous RNA
DN: Diabetic nephropathy
GEO: Gene expression omnibus
DEGs: Differentially expressed genes
PPI: Protein–protein Interaction
miRNA: MicroRNA
ROC: Receiver operating characteristic
GFR: Glomerular filtration rate
NEAT1: Nuclear enriched abundant transcript-1
XIST: X-inactive specific transcript
ncRNAs: Non-coding RNAs
IncRNAs: Long non-coding RNAs
CircRNAs: Circular RNAs
GSEA: Gene set enrichment analysis
GO: Gene ontology
KEGG: Kyoto encyclopedia of genes and genomes
PCA: Principal component analysis
UMAP: Uniform manifold approximation and projection
NES: Normalized enrichment score
FDR: False discovery rates
MCODE: Minimal common oncology data elements
MCC: Maximal clique centrality
DMNC: Density of maximum neighborhood component
MNC: Maximum neighborhood component
AUC: Areas under the curve
ECM: Extracellular matrix
MMPs: Matrix metalloproteinases
